# Low cuticle deposition rate in ‘Apple’ mango increases elastic strain, weakens the cuticle and increases russet

**DOI:** 10.1371/journal.pone.0258521

**Published:** 2021-10-13

**Authors:** Thomas O. Athoo, Bishnu P. Khanal, Moritz Knoche

**Affiliations:** Institute for Horticultural Production Systems, Leibniz-University Hannover, Hannover, Germany; Bahauddin Zakariya University, PAKISTAN

## Abstract

Russeting compromises appearance and downgrades the market value of many fruitcrops, including of the mango cv. ‘Apple’. The objective was to identify the mechanistic basis of ‘Apple’ mango’s high susceptibility to russeting. We focused on fruit growth, cuticle deposition, stress/strain relaxation analysis and the mechanical properties of the cuticle. The non-susceptible mango cv. ‘Tommy Atkins’ served for comparison. Compared with ‘Tommy Atkins’, fruit of ‘Apple’ had a lower mass, a smaller surface area and a lower growth rate. There were little differences between the epidermal and hypodermal cells of ‘Apple’ and ‘Tommy Atkins’ including cell size, cell orientation and cell number. Lenticel density decreased during development, being lower in ‘Apple’ than in ‘Tommy Atkins’. The mean lenticel area increased during development but was consistently greater in ‘Apple’ than in ‘Tommy Atkins’. The deposition rate of the cuticular membrane was initially rapid but later slowed till it matched the area expansion rate, thereafter mass per unit area was effectively constant. The cuticle of ‘Apple’ is thinner than that of ‘Tommy Atkins’. Cumulative strain increased sigmoidally with fruit growth. Strains released stepwise on excision and isolation (*ε*_exc+iso_), and on wax extraction (*ε*_extr_) were higher in ‘Apple’ than in ‘Tommy Atkins’. Membrane stiffness increased during development being consistently lower in ‘Apple’ than in ‘Tommy Atkins’. Membrane fracture force (*F*_max_) was low and constant in developing ‘Apple’ but increased in ‘Tommy Atkin’. Membrane strain at fracture (*ε*_max_) decreased linearly during development but was lower in ‘Apple’ than in ‘Tommy Atkins’. Frequency of membrane failure associated with lenticels increased during development and was consistently higher in ‘Apple’ than in ‘Tommy Atkins’. The lower rate of cuticular deposition, the higher strain releases on excision, isolation and wax extraction and the weaker cuticle account for the high russet susceptibility of ‘Apple’ mango.

## Introduction

Russeting of the skin compromises the appearance of many fruitcrop species, including of mango. Although the problem is mostly cosmetic, russeted fruits are usually excluded from high-end and export markets because of their unattractive, blotchy, dull-brown, appearance. Microscopic cracks (microcracks) in the cuticle are the first microscopic signs of russeting. Microcracks impair the barrier function of the cuticle. The consequences are: (1) an increased incidence of fruit rots [1] and (2) an unrestricted water movement into [2] and out of the fruit [3, 4]. Unrestricted water ingress can lead to bursting of epidermal cells and, eventually, to skin macrocracks that propagate deep into the flesh. Unrestricted water egress leads to increased postharvest water (weight) loss and, eventually, to skin shrivel. In a healthy growing fruit, the cuticle’s impaired barrier function, initiates the differentiation of a periderm which partially or wholly restores the barrier functions of the fruit skin. The first stage in the development of a periderm is for the cells of the hypodermis to become meristematic (a phellogen), these dividing cells then form stacks of waterproof (suberized) cork cells (the phellem). It is the suberized cell walls of the phellem that are responsible for the rough, dull-brown appearance of a russeted fruit.

The development of russeting is triggered by environmental factors including surface moisture and high humidity [4–6]. Large differences in susceptibility to russeting occur between species and, within species, between cultivars. Among Kenyan mango cultivars, cv. ‘Apple’ is highly susceptible to russeting, whereas cv. ‘Tommy Atkins’ is not. Due to its unattractive russeted appearance, the marketing of ‘Apple’ mango can be difficult.

Stress/strain relaxation analysis of the fruit skin is a tool that provides useful insight into the causal relationships underlying the formation of microcracks [7]. In this analysis, stress is removed stepwise, while the resulting strain releases are monitored [8]. The total area strain (*ε*_total_) represents the increase in surface area during fruit growth. This is partitioned into various component strains by stepwise removal of the stresses and monitoring the resulting strain releases. The component stresses leading to *ε*_total_ include the strain released on excision of an epidermal segment (*ε*_exc_), the strain released on (enzymatic) isolation of the cuticle (*ε*_iso_) and that released on extraction of the cuticular wax (*ε*_extr_). The remaining or residual strain (*ε*_resid_) may be calculated as the difference between the *ε*_total_ minus the sum of *ε*_exc_, *ε*_iso_, and *ε*_extr_. The residual strain *ε*_resid_ is irreversible, and it remains associated with the extracted cuticular membrane. In general, the buildup of elastic strain is considered not to be critical, provided the overall thinning of the cutin matrix is prevented by the continuous deposition of new cutin beneath it, and of wax within it, as its area expands.

The objective of this work was to understand the basis for the high susceptibility of ‘Apple’ mangoes to russeting. A better understanding is helpful in developing suitable countermeasures to reduce or prevent russeting in this cultivar. We focus on fruit growth, skin anatomy, cuticle deposition, stress/strain relaxation analysis and the mechanical properties of the cuticle. The russeting non-susceptible mango cultivar ‘Tommy Atkins’ served for comparison.

## Materials and methods

### Plant materials

‘Apple’ and ‘Tommy Atkins’ mangoes (*Mangifera indica* L.) were grafted on local, unclassified rootstocks. Fruit were harvested from a commercial orchard located in Mwala, Kenya (1°19’S, 37°26’E). The orchard was managed conventionally using local integrated crop management programs. Permission to sample fruit was given by the owner of the orchard Mr. and Mrs. Musyoka.

### Fruit growth

The time course of fruit growth was established first. Fruits were sampled every 1–3 weeks from 41 to 159 days after full bloom (DAFB). Fruits were weighed (Sartorius Pro 32/34F micro scales, Sartorious AG, Göttingen, Germany) and their length, and two orthogonal diameters were measured using calipers (CD-30PK; Mitutoyo, Kawasaki/Kanagawa, Japan). Fruit surface area was calculated assuming spherical shape. A sigmoid regression model was fitted through a plot of surface area vs. time. From this model, the rate of surface area growth (cm^2^ d^-1^) was calculated from the first derivative.

The relationships between fruit diameter, fruit surface area and fruit mass were established. Briefly, three fruit per cultivar were sampled every 2 to 3 weeks and weighed (Sartorius Pro 32/34F). Calibrated digital photographs were taken from two orthogonal aspects (Lumix DMC-G80; Panasonic Corporation, Osaka, Japan). The three orthogonal fruit diameters were quantified using image analysis (ImageJ 1.53P; National Health Institute, Bethesda, MD, USA). Fruit were then peeled, the peels flattened between two glass plates and digital photographs taken (Lumix DMC-G80; Panasonic Corporation). Peel area was quantified using image analysis (ImageJ 1.53P).

**Fig 1:**
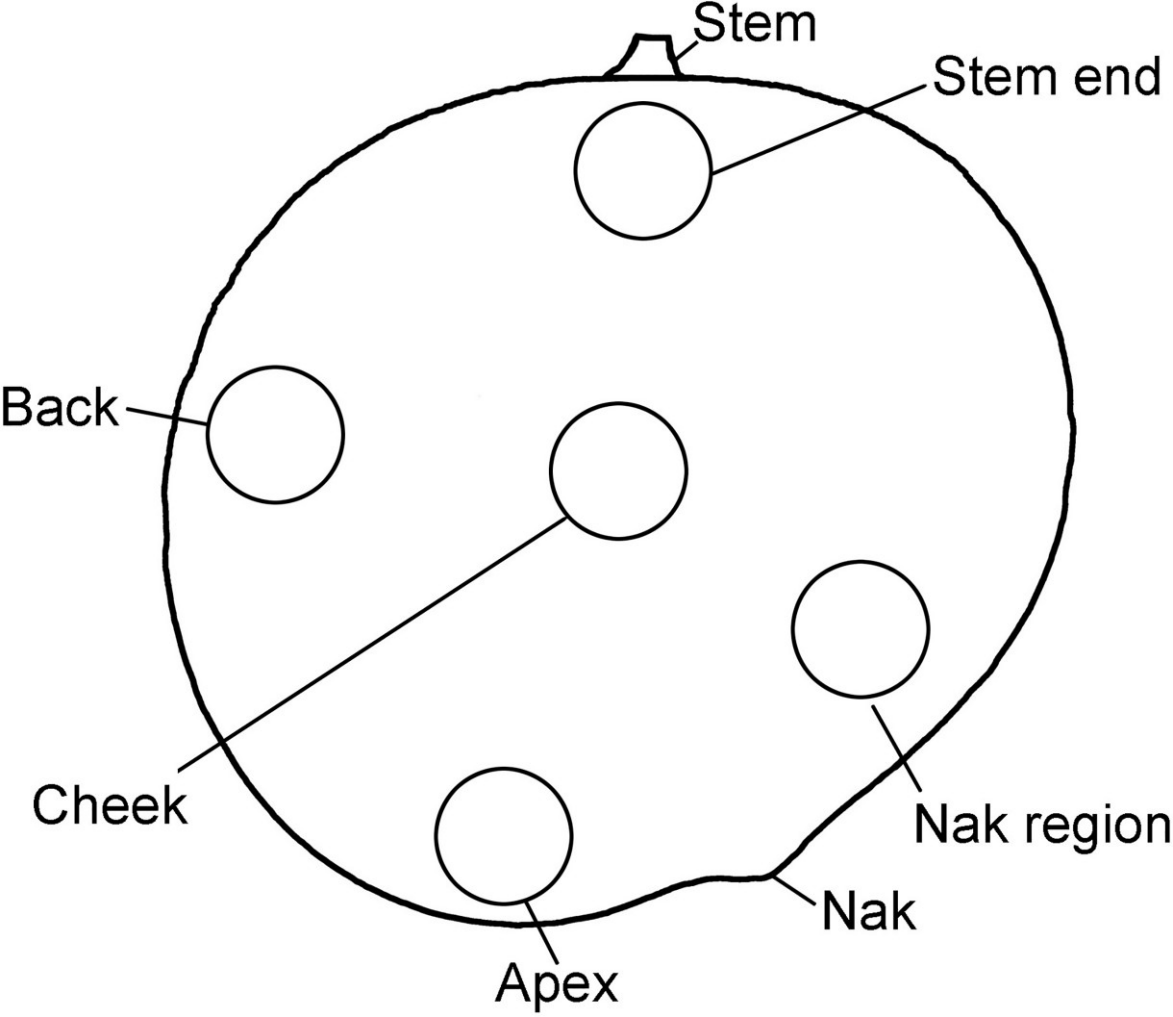
Sketch of mango fruit showing the different regions of the fruit surface sampled. These regions include stem end, cheek, apex, back and nak.

To obtain information on the spatial distribution of the skin area increase over the whole fruit surface, a set of fruit was tagged. A square pattern of four dots of a non-phytotoxic white silicon rubber (RTV 744; Dow Corning, Midland, MI, USA) was printed in five different regions on the fruit surface. The regions were: stem end, cheek, apex, back and nak (see Fig 1). The dot pattern was photographed at regular intervals (Lumix DMC-G80; Panasonic) and the area ‘enclosed’ by the dots quantified (ImageJ 1.53P). When necessary, dots were reapplied. We calculated the relative area growth rate (cm^2^ d^-1^) from a sigmoid curve fitted through a plot of ‘enclosed’ surface area vs. time for each region.

### Anatomy

Tissue blocks were excised from the fruit skin and the outer flesh, and incubated in Karnovsky fixative. These were stored in a cold room until use [9]. For processing, blocks were removed from Karnovsky solution and thoroughly rinsed with deionized water. Thin cross-sections of skin tissue were made in tangential periclinal direction in the cheek region of the fruit. The sections were stained for 5–8 min using 0.1% w/v calcofluor white (fluorescence brightener 28; Sigma-Aldrich Chemie, Munich, Germany), mounted on a microscope slide and transferred to the stage of a fluorescence microscope (BX-60; Olympus Europa Holding GmbH, Hamburg, Germany). Sections were examined under ultraviolet (UV) light (filter U-MWU 330–385 nm excitation, ≥420 nm emission; Olympus Europa) and calibrated images taken at ×20 magnification using a digital camera (DP73, Olympus Europa). The number of cells and cell dimensions (periclinal and anticlinal diameters) of the epidermal and three hypodermal cell layers were measured. Preliminary investigations established that epidermal and hypodermal cells are isodiametric in the tangential plane of the fruit as indexed by the absence of significant differences in their periclinal cell dimensions. There was no clear layering of hypodermal cells in either cultivar. We therefore measured the dimensions of all cells in each image (image size 349 × 262 μm) at 30, 50 and 70 μm depths from the surface of the cuticle. The periclinal area of a cell was calculated as the square of the cell’s periclinal diameter, the cell’s anticlinal aspect ratio as cell anticlinal diameter divided by the cell periclinal diameter. The number of cells per unit fruit surface area was counted.

### Lenticel density and area determination

The epidermal segments (ES) were excised from the stem end, cheek, apex, back and nak regions using a biopsy punch (8 mm diameter). The ES were photographed (Lumix DMC-G80; Panasonic) and the number of lenticels per disc counted (ImageJ 1.53P). Lenticel density was established by dividing the number of lenticels per ES by the area of the ES. The number of replicates was 30.

The areas of individual lenticels were quantified using enzymatically isolated cuticles. Lenticels were viewed under bright incident light using a binocular microscope (Leica MZ10F; Leica Microsystems, Wetzlar, Germany). Calibrated digital photographs were taken (Olympus DP 71; Olympus Europa). The areas of three to four lenticels per CM were measured using image analysis (cellSens version 1.7.1.; Olympus Europa). The number of replicates was 60 lenticels from 10 fruits.

### Cuticle deposition

Cuticles were isolated enzymatically. Briefly, ES were excised from the cheek region using a biopsy punch (8 or 10 mm diameter; Kai Europe, Solingen, Germany) at regular time intervals. The ES were incubated in an isolation medium containing pectinase (90 ml l^-1^; Panzym Super E flüssig, Novozymes A/S, Krogshoejvej, Bagsvaerd, Denmark), cellulase (5 ml l^-1^; Cellubrix L; Novozymes A/S) [10]. The pH was adjusted to 4.0 using NaOH. NaN_3_ was added at a final concentration of 30 mM to suppress microbial growth. The solution was refreshed regularly until the cuticular membrane (CM) separated from adhering cellular debris. The CM were cleaned using a camel-hair brush, then rinsed at least 5-times using deionized water.

For mass determination, CMs were dried at 40°C (Memmert 100–800; Memmert, Schwabach, Germany) and then weighed (CPA2P; Sartorius). Wax was extracted in a Soxhlet apparatus using chloroform: methanol (1:1 v/v CHCl_3_/MeOH) for a minimum of 2 h. The dewaxed cuticular membranes (DCMs) were dried overnight at 40°C (Memmert 100–800) and weighed. The CM, DCM and wax masses per unit area were calculated. The number of replicates was 10–20.

### Quantifying the areas of ES, CM and DCM

The ES were photographed (Lumix DMC-G80; Panasonic) and their areas quantified by image analysis (ImageJ 1.53P). Following enzymatic isolation, the fully-hydrated CMs were again photographed using a binocular microscope (Wild M10; Leica Microsysteme; camera DP71, Olympus Europa). Before dewaxing, a square pattern of holes was punched into the center of each CM disc and the hole pattern photographed. This was necessary because the DCMs curled up following extraction and it became impossible to flatten them. Two orthogonal diameters were measured on each ES or CM disc. For the DCM discs the areas enclosed by the hole patterns were calculated.

### Calculating apparent and cumulative strains

The developmental time course of change in apparent (ε′, %) and cumulative (ε, mm^2^mm^-2^) strain of the skin was calculated as the strain released upon excision, and upon isolation, and upon wax extraction [7]. The apparent strains released after excision and isolation of the CM ($\varepsilon_{exc+iso}^{\prime }$) and that released after dewaxing ($\varepsilon_{extr}^{\prime }$) were calculated using Eq 1 and Eq 2 respectively:

$$\varepsilon_{exc+iso}^{\prime }=\frac{A^{i}-A_{CM}^{i}}{A_{DCM}^{i}}\times 100$$ (1)

and

$$\varepsilon_{extr}^{\prime }=\frac{A_{CM}^{i}-A_{DCM}^{i}}{A_{DCM}^{i}}\times 100$$ (2)

In these equations, *A*^*i*^ represents the area of the skin disc before excision; $A_{CM}^{i}$ is the area of the isolated CM disc and $A_{DCM}^{i}$ is the area of an entire DCM disc. The latter was calculated from the square pattern of holes in the DCM. The sum of both these strains represents the total apparent strain ($\varepsilon_{total}^{\prime }$). As pointed out earlier, the area of the extracted CM disc at the particular time of sampling (t = i) serves as the basis of the calculation of apparent strains [7]. Any irreversible strain(s) that occur during growth of the carpel to the fruitlet at the time of sampling and that remain associated with the extracted CM discs are not accounted for.

These pitfalls are avoided when calculating the absolute cumulative strain (ε, mm^2^mm^-2^) of developing fruit [7]. Here the increase in fruit surface area with time is accounted for. In this analysis, the total cumulative strain (*ε*_total_) is partitioned into several component strains. The total cumulative strain (*ε*_total_) at time *t* = *i* was estimated as the change in surface area (Δ*A*^*i*^) relative to the initial surface area (*A*_0_) at time *t* = 0 (Eq 3). As discussed earlier [7], it is impractical to define the starting surface area and time *ab initio* (i.e., the differentiation of the carpel within a bud). We therefore defined the surface area at the time of initiation *A*_0_ somewhat arbitrarily as about 1 cm^2^ and calculated the total cumulative strain from Eq 3 [7].


$$\varepsilon_{total}=\frac{A_{total}^{i}-A_{0}}{A_{0}}$$ (3)

In this equation, $A_{total}^{i}$ represents the fruit surface area at a particular sampling time (*t* = *i*).

The cumulative strains released after excision and CM isolation (*ε*_exc+iso_) and after dewaxing (*ε*_extr_) were calculated as the differences in areas of the ES disc before excision (*A*^*i*^), and of the isolated CM (*A*_CM_), and of the extracted DCM (*A*_DCM_) (Eqs 4 and 5). Thus, *A*^*i*^ equals the cross-sectional area of the biopsy punch corrected for fruit curvature. The *A*_CM_ and *A*_DCM_ were determined at each sampling time and then calculated as the *A*_CM_ and *A*_DCM_ on a whole-fruit basis at a particular time (*t* = i). This calculation assumes the amount of strain release is uniform across the entire fruit surface.


$$\varepsilon_{exc+iso}=\frac{A_{total}^{i}-A_{CM}}{A_{0}}$$ (4)

$$\varepsilon_{extr}=\frac{A_{CM}-A_{DCM}}{A_{0}}$$ (5)

The strain that remains after dewaxing (*ε*_resid_) is fixed mostly by the cutin matrix and is calculated from Eq 6 as follows:

$$\varepsilon_{resid}=\frac{A_{DCM}-A_{0}}{A_{0}}$$ (6)

As explained earlier [7], the total cumulative strain (*ε*_total_) represents the sum of these individual component strains because all were calculated relative to the same base (*A*_0_) (Eq 7):

$$\varepsilon_{total}=\varepsilon_{exc+iso}+\varepsilon_{extr}+\varepsilon_{resid}.$$ (7)

Of the three component strains, *ε*_extr_ and *ε*_resid_ represent the plastic strain (*ε*_plastic_), whereas the *ε*_exc+iso_ is the elastic strain.

### Uniaxial tensile tests

Strips (5 mm wide) were excised from the CM isolated between 55 and 159 DAFB using parallel-mounted razor blades. Strips were mounted in a cardboard frame made from masking tape to prevent unintentional strain during handling. Strips were hydrated overnight by incubation in deionized water. Thereafter, strips were mounted in a universal material testing machine (clamping distance *l*_0_ = 10 mm) (Z 0.5; Zwick/Roell, Ulm, Germany) equipped with a 10 N force transducer (KAP-TC; Zwick/Roell). Frames were cut open and the test initiated at a strain rate of 0.25 mm min^-1^.

Stiffness (*S*) was calculated as the maximum slope of the force (*F*, N) vs. strain (*ε*_tensile_, %) diagram. Uniaxial strain was calculated as the ratio of the applied strain (Δ*l*) divided by the length of the relaxed sample, i.e., the clamping distance *l*_0_ (mm) and multiplied by 100. The maximum force (*F*_max_) and maximum strain (ε_max_, %) represent the force and strain recorded at failure. Following testing, the fracture site was inspected on each specimen to identify whether or not the fracture was associated with a lenticel.

### Data analysis and presentation

Data are presented as means and standard errors (SE). Where not visible, the SEs are smaller than the data symbols. Data were analyzed using analysis of variance with R statistical software (R version 4.0.3; R Foundation for Statistical Computing, Vienna, Austria) and SAS (SAS Institute, Cary, NC, USA). Means were separated using Turkey’s studentized range test (α = 0.05). Regression analyses were conducted in R (R version 4.0.3) and Sigma Plot (version 12.5; Systat Software, San Jose, CA, USA). Significances of regression equations at the 5, 1 and 0.1% levels are indicated by *, ** and ***, respectively.

## Results

**Fig 2:**
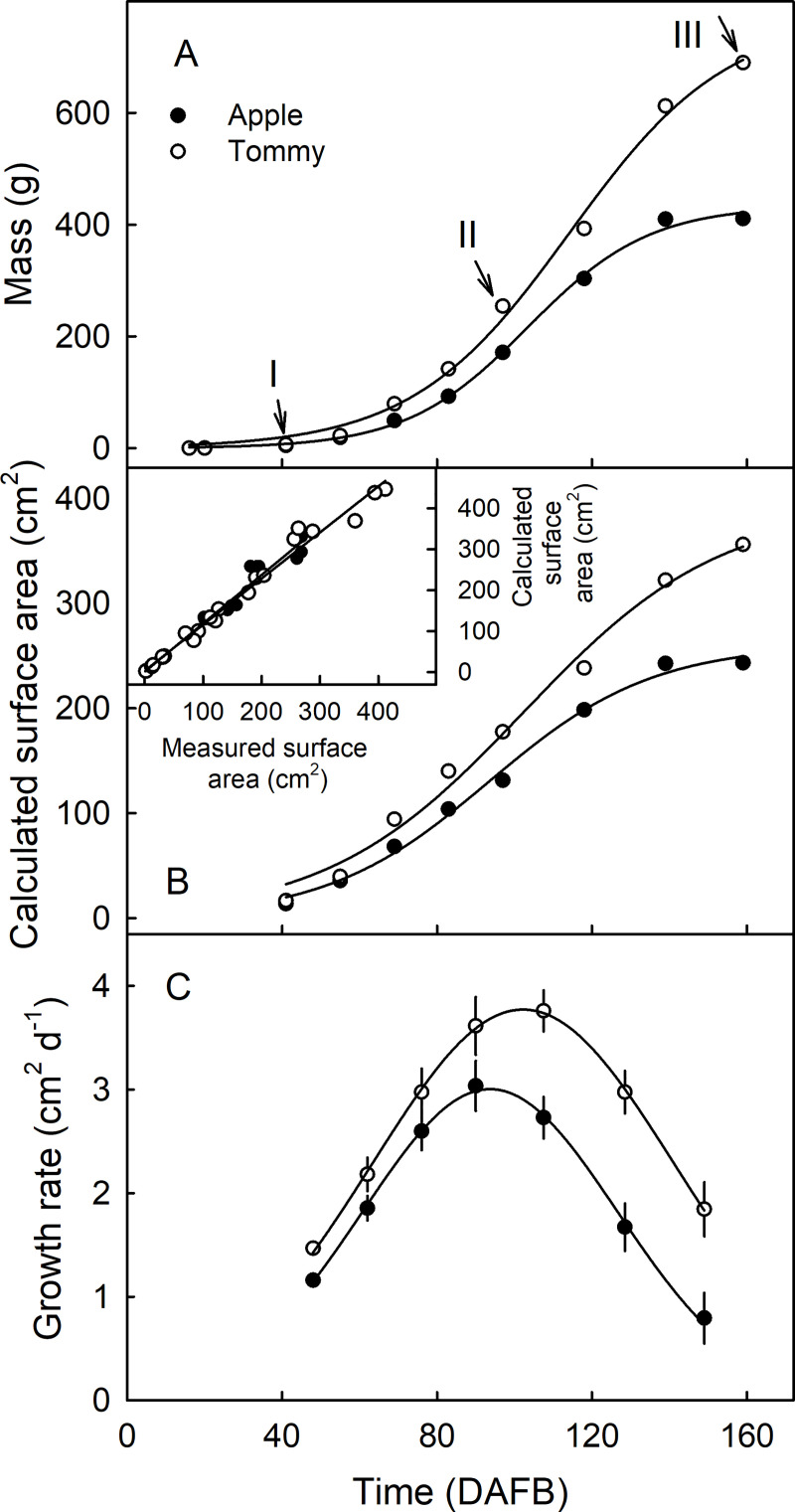
Time course of change in mass (A), calculated surface area (B) and growth rate (C) of ‘Apple’ and ‘Tommy Atkins’ mango. Inset in B: Plot of measured and calculated surface area of the fruit peel. The area was calculated assuming a spheroid shape. The x-axis scale is in days after full bloom (DAFB). Arrows indicate the sampling dates for histological examination. Data represent means ± SE. The number of individual fruit replicates was 10–20.

Fruit mass and surface area increased with time in a sigmoid pattern in both cultivars (Fig 2A and 2B). Fruits of ‘Apple’ had lower masses, lower surface areas and lower growth rates than fruits of ‘Tommy Atkins’ (Fig 2A). In ‘Apple’, the area growth rate was at maximum of 3.03 cm^2^ d^-1^ at 94 DAFB. On the other hand, ‘Tommy Atkins’ had a maximum growth rate of 3.85 cm^2^ d^-1^ at about 103 DAFB (Fig 2C). There was a strong linear, positive correlation (r^2^ = 0.98***) between fruit surface area measured on excised peels and that calculated from fruit dimensions (Fig 2B, inset).

**Fig 3:**
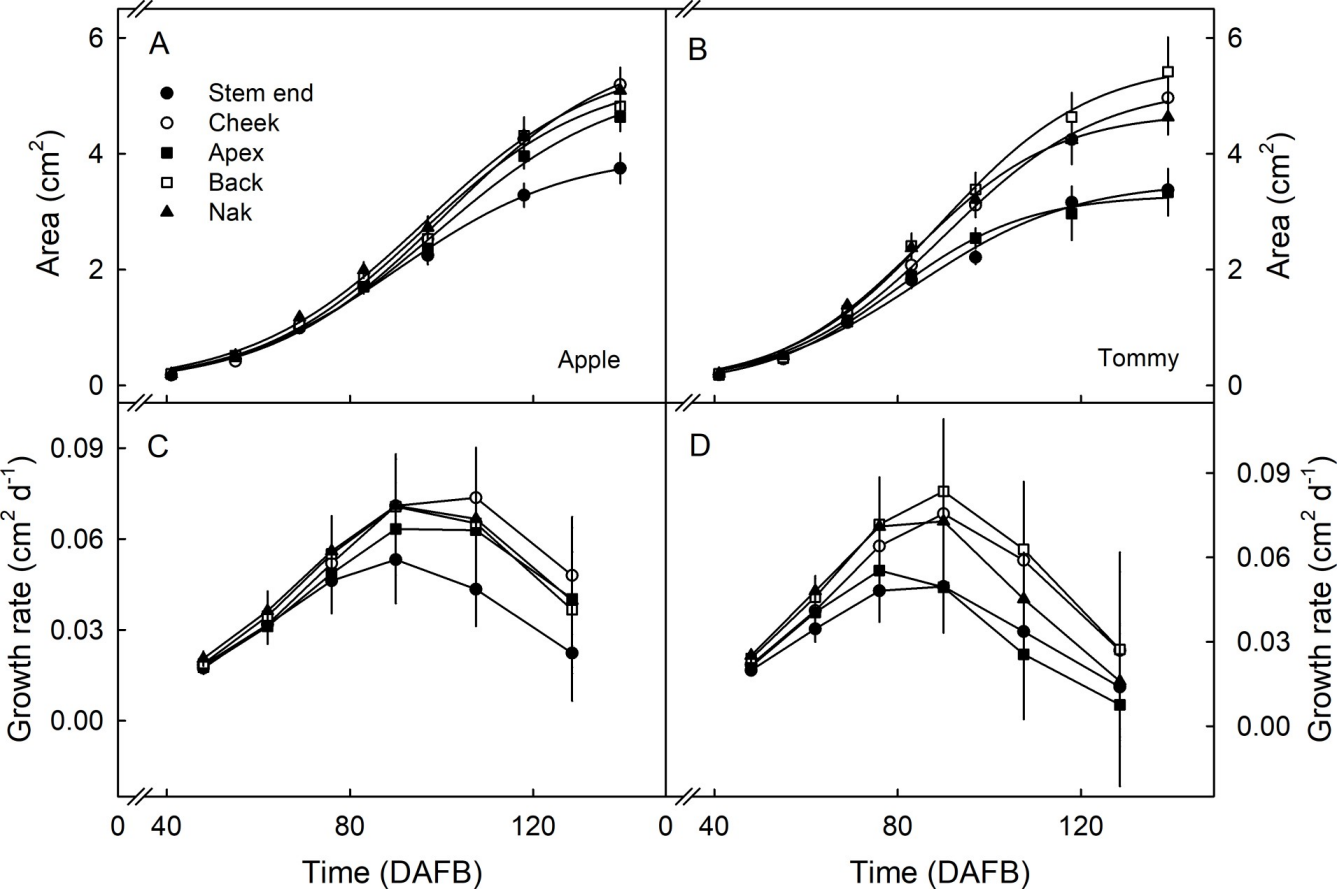
Time course of change in area (A,B) and growth rate (C,D) of selected regions of the fruit surface of ‘Apple’ (A,C) and ‘Tommy Atkins’ (B,D) mango. A square pattern of dots of white silicon rubber was printed in the stem end, the cheek, the apex, the back or the nak region of the fruit surface at 41 days after full bloom (DAFB) and the expansion of the dot pattern monitored. For location of the regions on the fruit surface see Fig 1. Data represent means ± SE. The number of replicates was 16–30.

The growths in the different regions of the fruit surface measured using the dot pattern also increased with time in a sigmoid pattern (Fig 3A and 3B). There were no significant differences in cumulative increases in surface area or in growth rates between these regions in either cultivar, during early fruit development (Fig 3).

**Fig 4:**
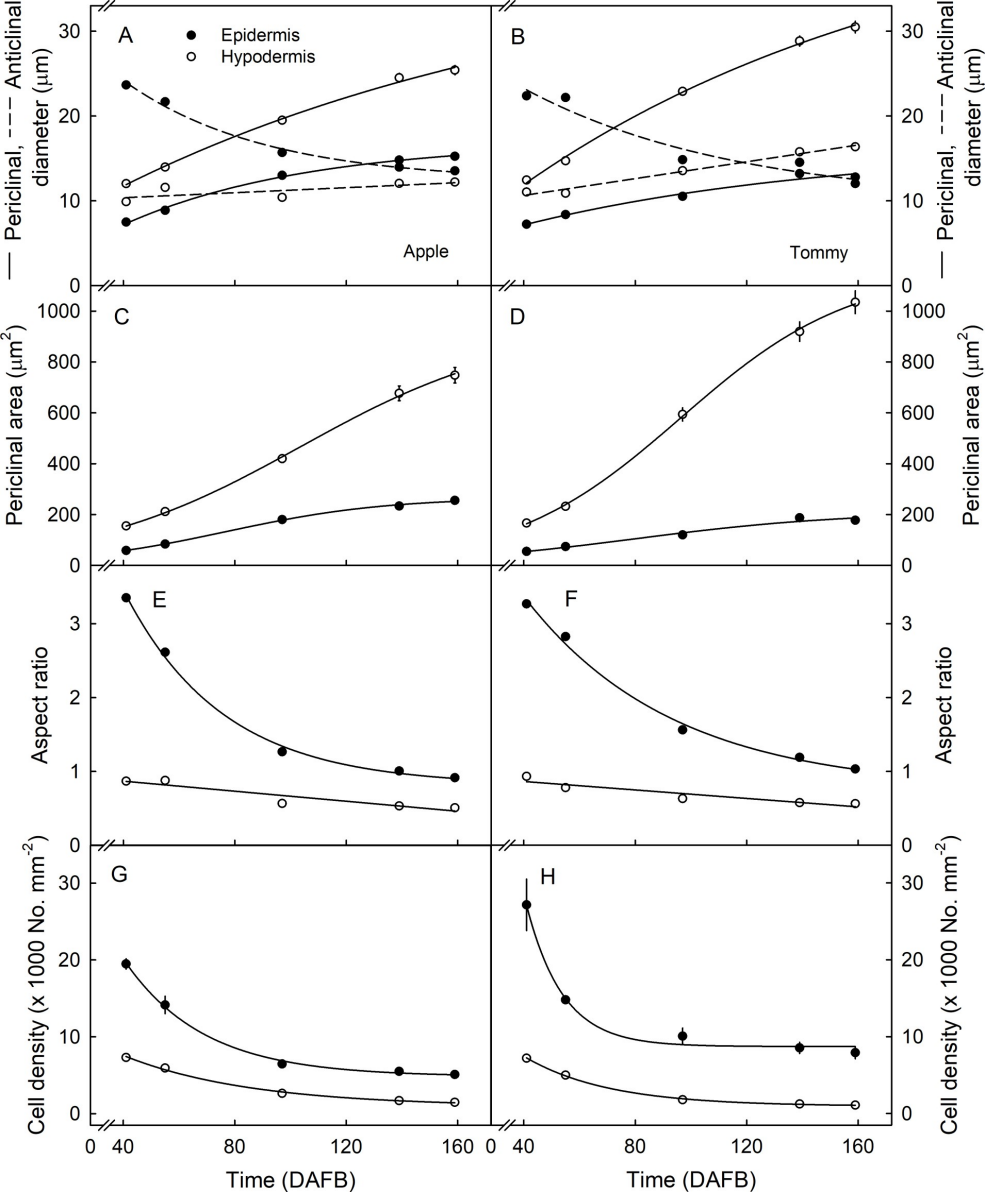
Developmental time course of change in anticlinal and periclinal diameters (A,B), periclinal area per cell (C,D), anticlinal aspect ratio (E,F) and number of epidermal and hypodermal cells per unit fruit surface area (G,H) of ‘Apple’ (A,C,E,G) and ‘Tommy Atkins’ (B,D,F,H) mango. The anticlinal aspect ratio was calculated as the anticlinal diameter divided by the periclinal diameter. The periclinal surface area of a cell was calculated as the square of cell periclinal diameter. The x-axis scale is in days after full bloom (DAFB). Data represent means ± SE. The number of individual fruit replicates was 10.

Periclinal diameters of epidermal and hypodermal cells increased with development, whereas the anticlinal diameter of epidermal cells decreased in both cultivars. The anticlinal diameters of hypodermal cells in ‘Apple’ remained unchanged, but increased in ‘Tommy Atkins’ (Fig 4A and 4B). Epidermal cells were smaller than hypodermal cells. There was little difference in the dimensions of epidermal cells between ‘Apple’ and ‘Tommy Atkins’, but hypodermal cells had larger periclinal diameters in ‘Tommy Atkins’ than in ‘Apple’ (Fig 4A and 4B). Epidermal cells had markedly smaller periclinal areas than the hypodermal cells. There was little difference between the two cultivars. Periclinal areas of hypodermal cells increased in a sigmoid pattern with time. They were smaller in ‘Apple’ than in ‘Tommy Atkins’ (Fig 4C and 4D). Epidermal cells changed their anticlinal aspect ratios from ‘portrait’ to ‘square’ in both ‘Apple’ and ‘Tommy Atkins’. Compared to the epidermal cells, the change in anticlinal aspect ratio of the hypodermal cells was much smaller. The hypodermal cells were nearly isodiametric in the anticlinal plane during early development, but elongated to ‘landscape’ towards maturity (Fig 4E and 4F). There was no significant difference in cell number per unit surface area between ‘Apple’ and ‘Tommy Atkins’ (Fig 4G and 4H).

**Fig 5:**
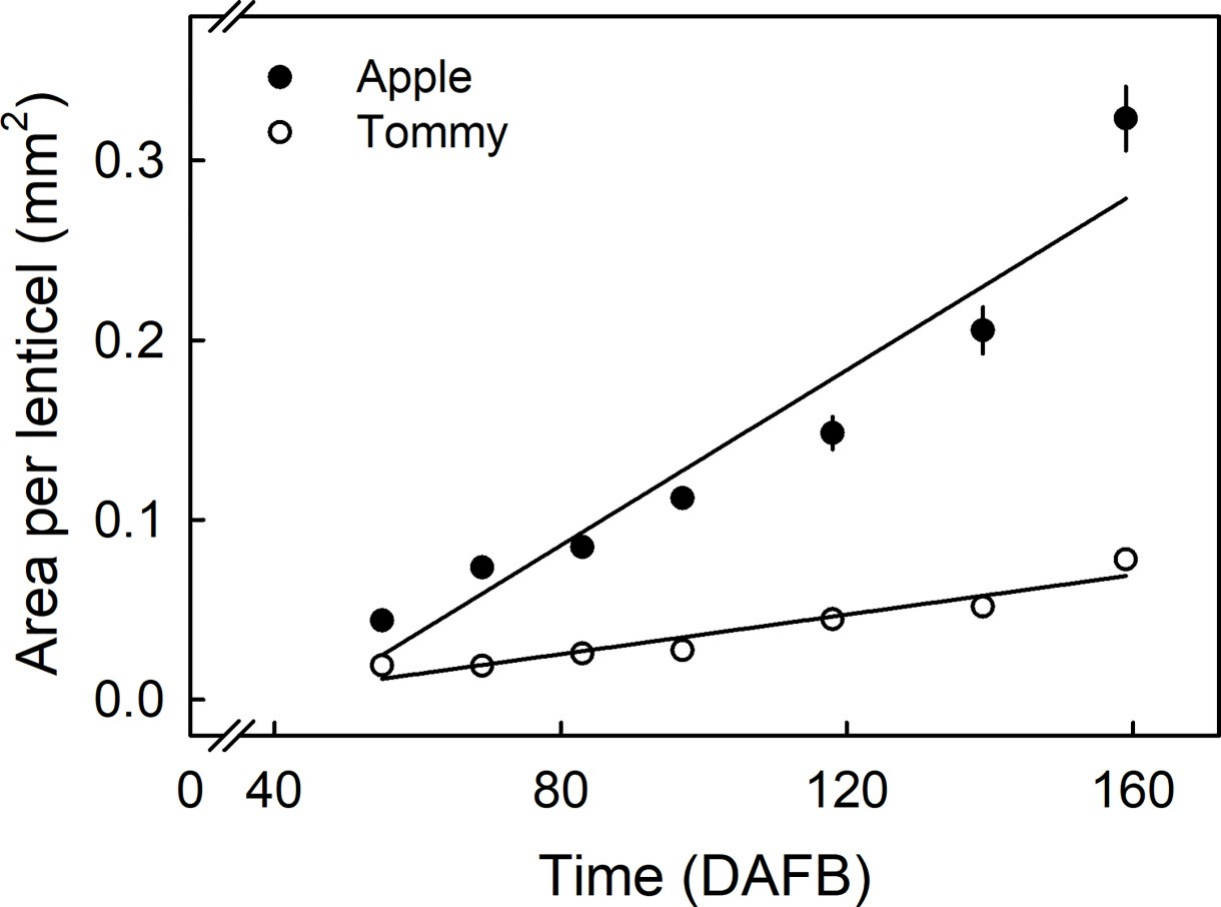
Developmental time course of change in area of lenticels in the cheek region of ‘Apple’ and ‘Tommy Atkins’ mango. The x-axis scale is in days after full bloom (DAFB). Data represent means ± SE. The number of replicates was 60 lenticels.

Lenticel density decreased with time during development and was generally lower in ‘Apple’ than in ‘Tommy Atkins’ (Table 1). In both cultivars, the apex had the highest lenticel density while the cheek had the lowest (Table 1). The area per lenticel increased linearly during development (Fig 5). Fruits of ‘Apple’ mango consistently had larger lenticels than ‘Tommy Atkins’.

**Table 1:** Lenticel density established from epidermal section of ‘Apple’ and ‘Tommy Atkins’ mango fruit skin.

Cultivar	Time (DAFB)	Lenticel density (No. mm^-2^)					
		Apex	Cheek	Stem End	Back	Nak	Mean _Cultivar_
Apple	78	0.45 ± 0.02 c ^a^	0.18 ± 0.01 a	0.26 ± 0.02 b	0.25 ± 0.02 b	0.14 ± 0.01 a	0.26 ± 0.01
	107	0.31 ± 0.02 d	0.08 ± 0.01 a	0.24 ± 0.01 c	0.16 ± 0.01 b	0.09 ± 0.01 a	0.18 ± 0.01
	152	0.28 ± 0.01 d	0.04 ± 0.00 a	0.19 ± 0.01 c	0.10 ± 0.01 b	0.09 ± 0.01 b	0.14 ± 0.01
Tommy Atkins	78	1.05 ± 0.06 c	0.38 ± 0.02 a	0.37 ± 0.03 a	0.62 ± 0.05 b	0.64 ± 0.05 b	0.62 ± 0.03
	107	0.73 ± 0.04 c	0.24 ± 0.02 a	0.23 ± 0.01 a	0.35 ± 0.02 b	0.30 ± 0.02 ab	0.37 ± 0.02
	152	0.40 ± 0.03 b	0.15 ± 0.01 a	0.21 ± 0.02 a	0.18 ± 0.01 a	0.18 ± 0.01 a	0.23 ± 0.01
Mean _Region_ ’Apple’		0.35 ± 0.01	0.10 ± 0.01	0.23 ± 0.01	0.18 ± 0.01	0.11 ± 0.01	0.19 ± 0.01
Mean _Region_ ’Tommy Atkins’		0.73 ± 0.02	0.26 ± 0.02	0.27 ± 0.02	0.38 ± 0.02	0.38 ± 0.02	0.40 ± 0.01
Mean _Region_		0.54 ± 0.01	0.18 ± 0.01	0.25 ± 0.01	0.28 ± 0.01	0.24 ± 0.01	

The ES were excised from the apex, cheek, stem end, back and nak regions of the same fruits at 78, 107 or 152 days after full bloom (DAFB). Data presented as means ± SE. The number of replicates was 30.

^a^ Main effect of cultivar, time (DAFB) and fruit region and their interaction significant in a three factorial ANOVA. Means therefore compared across the regions at each time (DAFB) and for each cultivar. Mean separation within rows by Tukey’s studentized range test, P < 0.05.

**Fig 6:**
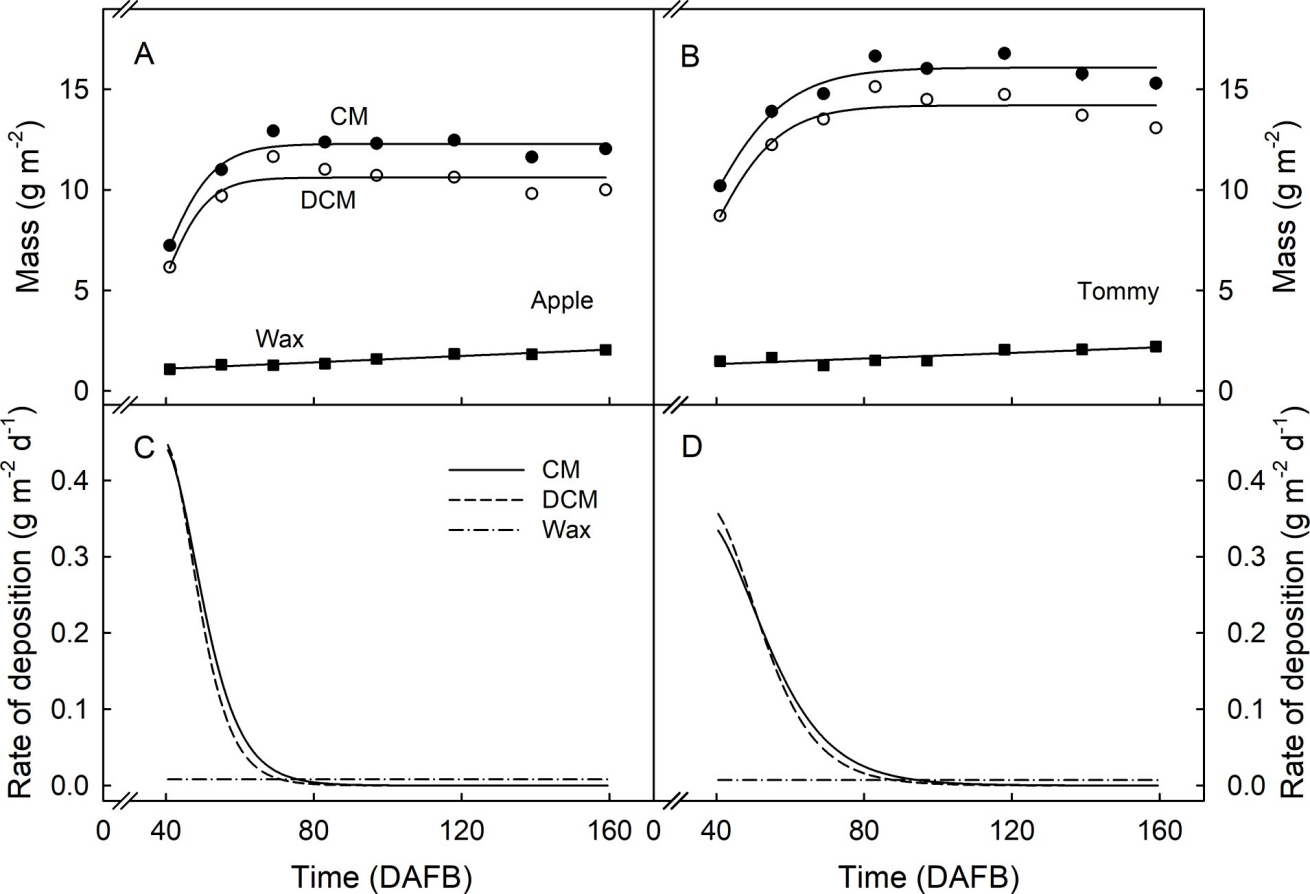
Developmental time course of change in mass of cuticular membrane (CM), dewaxed CM (DCM) and wax of ‘Apple’ (A) and ‘Tommy Atkins’ (B) mango. Rates of deposition of CM, DCM, and wax of ‘Apple’ (C) and ‘Tommy Atkins’ (D) mango. The rates of deposition were calculated as the first derivative of a regression equation fitted through a plot of CM, DCM or wax mass vs. time. The x-axis scale is in days after full bloom (DAFB). Data in Fig 6A and 6B represent means ± SE. The number of replicates was 33–35.

The deposition of CM and DCM was rapid during early development as indexed by a marked increase in mass per unit area. From about 69 DAFB (‘Apple’) and 83 DAFB (‘Tommy Atkins’) onwards, the mass of the CM and DCM per unit area remained constant indicating that the rate of deposition kept pace with surface area expansion (Fig 6A and 6B). The mass of wax per unit area increased slightly during development in both cultivars indicating that the wax deposition rate slightly exceeded that required to compensate for area expansion (Fig 6). In both cultivars, the rates of CM and DCM deposition decreased rapidly during early development but remained constant thereafter (Fig 6C and 6D).

**Fig 7:**
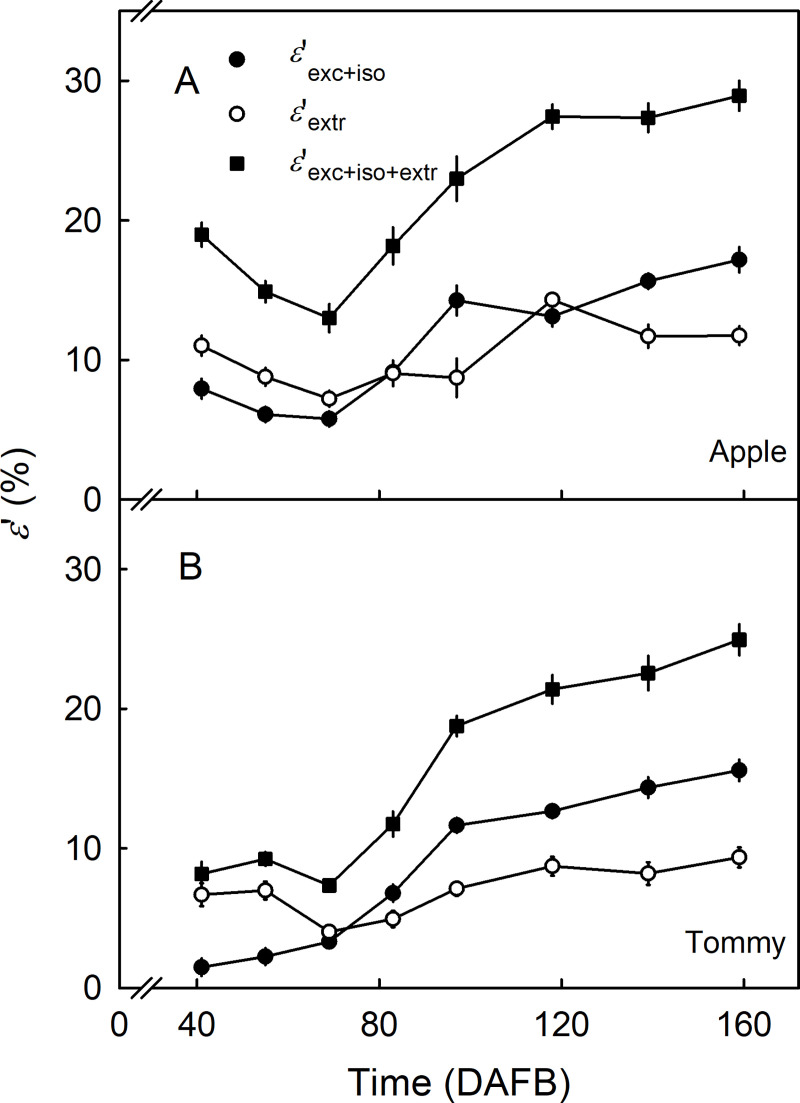
Time course of change in the apparent strain (*ε*′, %) in the cuticular membrane (CM) of ‘Apple’ (A) and ‘Tommy Atkins’ (B) mango. These strains were quantified as the releases after excision and isolation of the CM ($\varepsilon_{exc+iso}^{\prime }$), after wax extraction ($\varepsilon_{extr}^{\prime }$), and their sum ($\varepsilon_{exc+iso+extr}^{\prime }$). The x-axis scale is in days after full bloom (DAFB). Data represent means ± SE. The number of replicates was 10–20.

In both cultivars, the apparent strains released on excision and isolation ($\varepsilon_{exc+iso}^{\prime }$) and those released on wax extraction ($\varepsilon_{extr}^{\prime }$) increased as fruit surface area increased, particularly from 69 DAFB onwards when the fruit surface area began to increase rapidly. Up to 69 DAFB, $\varepsilon_{extr}^{\prime }$ tended to be larger than $\varepsilon_{exc+iso}^{\prime }$ in both cultivars (Fig 7). However, during later development, $\varepsilon_{exc+iso}^{\prime }$ generally exceeded $\varepsilon_{extr}^{\prime }$ (Fig 7). At maturity, the sum of the two ($\varepsilon_{exc+iso+extr}^{\prime }$) was significantly greater in ‘Apple’ (28.9 ± 1.1%) than in ‘Tommy Atkins’ (24.9 ± 1.1%) (Fig 7).

**Fig 8:**
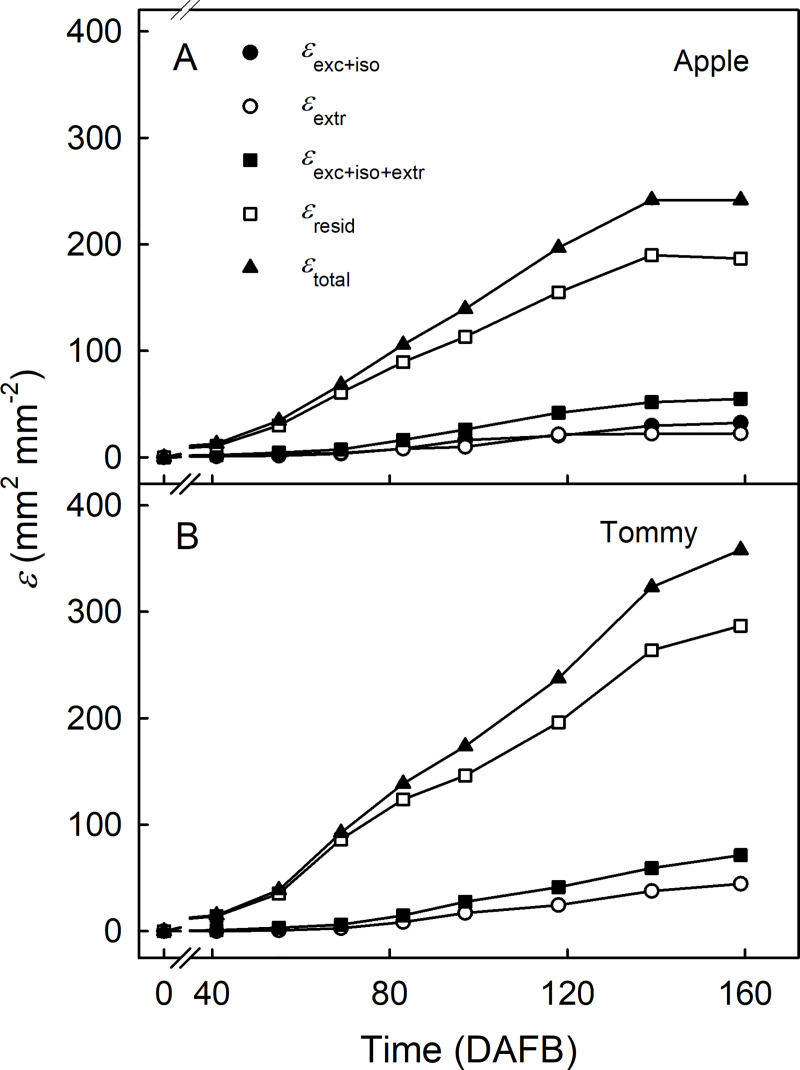
Developmental time course of change in total cumulative strain (*ε*_*total*_), strain released upon excision and isolation of the cuticular membrane (CM) (*ε*_*exc*+*iso*_), that released after the extraction of wax (*ε*_*extr*_), the sum of the two strains (due to wax and tissue) (*ε*_*exc*+*iso*+*extr*_), and the remaining strain (*ε*_*resid*_) of the skin of developing ‘Apple’ (A) and ‘Tommy Atkins’ (B) mango. The total cumulative strain of the CM (*ε*_*total*_) was estimated from the increase in surface area with time. The x-axis scale is in days after full bloom (DAFB). Data represent means ± SE. The number of replicates was 10–20.

Cumulative strain increased with fruit growth in a sigmoid pattern. In ‘Apple’ and ‘Tommy Atkins’, *ε*_resid_ accounted for most of the total cumulative strain (*ε*_total_) (Fig 8). The contributions to *ε*_total_ of strain due to excision and isolation (*ε*_exc+iso_), to wax extraction (*ε*_extr_) and their sum (*ε*_exc+iso+extr_) were significantly lower than the *ε*_resid_ (Fig 8).

**Fig 9:**
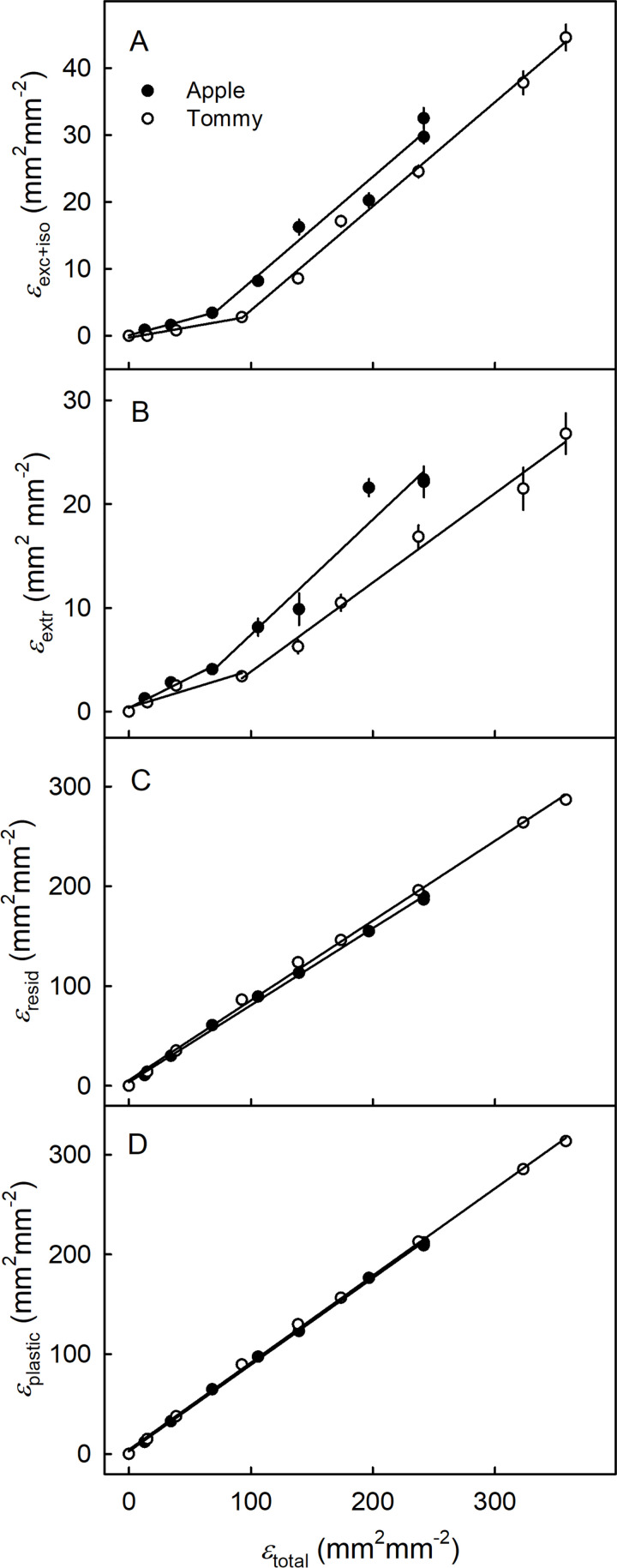
Cumulative strains released after excision and isolation (*ε*_exc+iso_) of the cuticular membrane (CM) (A), after wax extraction from the CM (*ε*_extr_) (B), the strain that remains in the dewaxed CM (*ε*_resid_) (C) and the sum of *ε*_extr_ and *ε*_resid_ (*ε*_plastic_) (D) plotted against the total cumulative strain (*ε*_total_) of the surface of developing ‘Apple’ and ‘Tommy Atkins’ mango. The *ε*_total_ was estimated from the increase in surface area with time relative to the surface area at bloom. The *ε*_resid_ was estimated as the difference between the total strain (*ε*_total_) and the strain due to the wax and tissue (*ε*_exc+iso+extr_). Data represent means ± SE. The number of replicates was 10–20.

Plotting *ε*_exc+iso_, *ε*_extr_, *ε*_resid_ and *ε*_plastic_ vs. *ε*_total_ revealed biphasic relationships for *ε*_exc+iso_ and *ε*_extr_ vs. *ε*_total_, but linear relationships for *ε*_resid_ and *ε*_plastic_ vs. *ε*_total_ (Fig 9). Initially, *ε*_exc+iso_ and *ε*_extr_ increased slowly with *ε*_total_. Beyond a breakpoint at *ε*_total_ about 70, the slope increased and *ε*_exc+iso_ and *ε*_extr_ increased more rapidly. The increases were higher in ‘Apple’ than in ‘Tommy Atkins’ (Fig 9A and 9B). In contrast, *ε*_resid_ increased linearly with *ε*_total_ and accounted for most of *ε*_total_ in both cultivars (Fig 9C and 9D).

**Fig 10:**
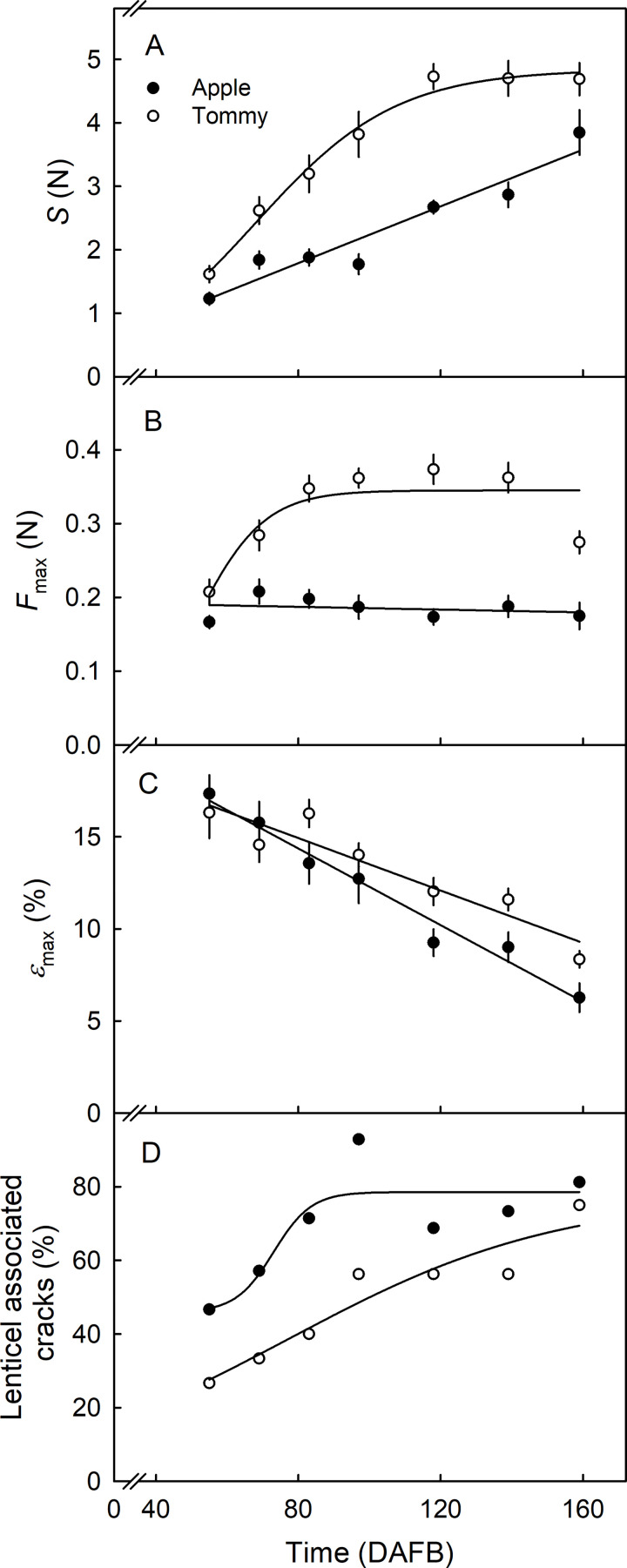
Developmental time course of change in stiffness (S) (A), maximum force (*F*_max_) (B) and strain at maximum force (*ε*_max_) (C) of the cuticular membrane (CM) of developing ’Apple’ and ’Tommy Atkins’ mango. (D) Frequency of occasions where failure was associated with lenticels. The x-axis scale is in days after full bloom (DAFB). Data in Fig 10A-10C represent means ± SE. The number of replicates was 13–16.

Stiffness (S) of the CMs increased during development but was consistently lower in ‘Apple’ than in ‘Tommy Atkins’ (Fig 10A). There was no significant change in the fracture force (*F*_max_) in ‘Apple’ during development, but *F*_max_ in ‘Tommy Atkins’ increased up to about 83 DAFB (Fig 10B). The value of *F*_max_ was significantly lower in ‘Apple’ than in ‘Tommy Atkins’ (Fig 10B). The strain at fracture (*ε*_max_) decreased linearly with development. The value of *ε*_max_ was lower in ‘Apple’ than in ‘Tommy Atkins’ (Fig 10C). The frequency of failure associated with lenticels increased during development and was consistently higher in ‘Apple’ than in ‘Tommy Atkins’ (Fig 10D).

## Discussion

This discussion focusses on (1) the relationship between fruit growth, cuticle deposition, and stress/strain relaxation and (2) the mechanical characteristics of the cuticle and their relationships to russet susceptibility.

### Stress/Strain relaxation behavior of the cuticle reflects fruit growth and cuticle deposition

The stress/strain relaxation analysis reveals several differences between ‘Apple’ and ‘Tommy Atkins’. First, total cumulative strain was greater in ‘Tommy Atkins’ due to its larger fruit and, hence, greater fruit surface area (Figs 2 and 8). Second, the relationships between *ε*_exc+iso_ and *ε*_total_ (Fig 9A) and between *ε*_extr_ and *ε*_total_ were biphasic in both cultivars (Fig 9B). During early development, there was little change in *ε*_exc+iso_ and *ε*_extr_ as *ε*_total_ increased. The increase in *ε*_total_ was primarily accounted for by an irreversible increase in *ε*_resid_ (Fig 9C). Mechanically, the increase in *ε*_resid_ resulted principally from a deposition of cutin. In *Malus* apple, the addition of cutin to the inner side (cell-wall side) of the cuticle on the expanding fruit surface resulted in the fixation of elastic strain and, thus, the development of a radial strain gradient within the cuticle [11]. The incorporation of wax into the cutin network also causes a small degree of ‘fixation’ of elastic strain (reversible) and, hence, also contributes to the plastic strain (irreversible) [12]. Our results indicate the relationships earlier identified in *Malus* apple, also occur in ‘Apple’ and ‘Tommy Atkins’ mangoes. Here, the ongoing deposition of CM fixes the reversible elastic strains by converting them into irreversible plastic strains in both ‘Apple’ and ‘Tommy Atkins’. Accordingly, when the rate of CM deposition decreased, approaching an asymptote at 59 DAFB (‘Apple’; Fig 6C) and 68 DAFB (‘Tommy Atkins’; Fig 6D), during the second phase, *ε*_exc+iso_ and *ε*_extr_ both increased at higher rates as *ε*_total_ also increased (Fig 9). While, from a practical point of view, *ε*_exc+iso_ is truly elastic and reversible, *ε*_extr_ is a plastic strain in the cuticle on an expanding surface. However, mechanically, this strain is also reversible, since the strained cutin polymer relaxes when the wax that blocks the relaxation *in vivo* is extracted *in vitro*. It is particularly interesting that the breakpoint in the above relationships occurs earlier in ‘Apple’, as indexed by the lower *ε*_total_, than in ‘Tommy Atkins’ (Fig 9). This observation is also consistent with the earlier breakpoint in CM deposition in ‘Apple’ as compared to ‘Tommy Atkins’. Furthermore, for any given value of *ε*_total_, *ε*_exc+iso_ and *ε*_extr_ were consistently higher in ‘Apple’ than in ‘Tommy Atkins’ (Fig 9). This is in line with the lower rate of cutin deposition in ‘Apple’ than in ‘Tommy Atkins’ (Fig 6A and 6B). These arguments demonstrate that growth and cuticle deposition are the primary determinants of cuticle stress and strain. Both processes account for the differences between ‘Apple’ and ‘Tommy Atkins’ identified in the stress/strain relaxation analysis.

### The cuticle of ‘Apple’ is mechanically weaker than that of ‘Tommy Atkins’

Uniaxial tensile tests revealed that the CM of ‘Apple’ is mechanically weaker than that of ‘Tommy Atkins’ (Fig 10). Stiffness, *F*_max_ and *ε*_max_ in ‘Apple’ CMs are all significantly lower than in ‘Tommy Atkins’ CMs. This implies that ‘Apple’ CMs fracture at lower force and lower strain than ‘Tommy Atkins’. The lower cutin mass (Fig 6A and 6B), the larger lenticels (Fig 5), and the greater strain release on excision of the ES and on isolation of the CM (Figs 7 and 9A) and on extraction of the CMs of ‘Apple’ (Figs 7 and 9B) may explain their relative weakness, compared with the CMs of ‘Tommy Atkins’ [13]. In addition, the presence of lenticels may also affect the mechanical strength of the CM. Microcracks in the CM are almost always initiated at a lenticel and these are more frequent in ‘Apple’ than ‘Tommy Atkins’ (Fig 5, Table 1). Also, as fruit surface area increases, lenticel area also increases, while the number of lenticels per unit area decreases (the total number of lenticels per fruit being constant). The increase in lenticel area occurs at a markedly higher rate in ‘Apple’ than in ‘Tommy Atkins’ (Fig 5). At maturity, lenticel area was about three-times greater in ‘Apple’ than in ‘Tommy Atkins’. The contribution of lenticels to the mechanical strength of mango skin is unknown. However, in the skins of grapes, the lenticels represent points of stress concentration and, hence, of weakness [14]. Furthermore, in *Malus* apple the cuticle-periderm boundary is the weak link, often causing skin strips to facture in this region when subjected to uniaxial tensile tests [15]. The larger lenticels of ‘Apple’ mango also imply a larger cuticle-periderm boundary and hence, a weaker skin. We observed cracking across or around lenticels in 70% and 59% of the CM strips of ‘Apple’ and ‘Tommy Atkins’, respectively (data not presented). This is consistent with the above hypothesis. Mechanically, the increase in area per lenticel in ‘Apple’ mango may also be interpreted as a mechanically weak periderm of the lenticel, that fails continuously during fruit expansion, thereby triggering a continuous formation of periderm.

The size, orientations and number of epidermal and hypodermal cells were generally similar in ‘Apple’ and ‘Tommy Atkins’ mangoes (Fig 4). In *Malus* apples, the more russet-resistant cultivars are characterized by having smaller and more compactly arranged epidermal and hypodermal cells [16]. Furthermore, the russet-resistant apple cultivars had smaller cells that were less variable in size than those of the more russet-susceptible cultivars [17]. However, there were no comparable and consistent differences between ‘Apple’ and ‘Tommy Atkins’ mangoes.

Lower rates of CM deposition and larger lenticels therefore predispose the ‘Apple’ mango skin to fracture when subjected to strain induced by normal growth, thereby accounting for its higher susceptibility to russeting. The cuticular fractures impair the barrier properties of the skin and trigger the formation of a periderm. This sequence of events is consistent also with the initiation of russeting at lenticels and the exacerbating effects of surface moisture on russeting in ‘Apple’ mango [3]. ‘Apple’ mango is not unique in this respect. Similar relationships have been identified in *Malus* apple [18–20]. Unlike susceptible ‘Apple’ mango, russeting in *Malus* apple is not initiated in the lenticels.

The trigger(s) that initiates the dedifferentiation in the hypodermis, the differentiation of a phellogen and the cell division in the phellogen to produce the phellem is not known. Potential candidates are a change in the O_2_ or CO_2_ concentrations in the internal atmosphere of the fruit or a local decrease (more negative) in water potential in the region surrounding the microcracks due to increase transpiration [20].

## Conclusion

Our findings indicate that the susceptibility to russeting of ‘Apple’ compared to ‘Tommy Atkins’ is due to the following sequence of events. Compared to the non-susceptible ‘Tommy Atkins’, ‘Apple’ mango has a lower rate of CM deposition which results in higher elastic strain. The high elastic strain serves to initiate cuticular microcracking, which is exacerbated by surface wetness. In this sequence of events, the different elasticity of the lenticels causes local stress concentrations in ‘Apple’ mango, but not in ‘Tommy Atkins’ mango. Microcracking weakens the cuticle, that also now has impaired barrier properties as indexed by increased transpiration [3]. The impaired barrier properties are likely to be the trigger for periderm formation that causes the discoloration known as russeting on the surface of russet-susceptible ‘Apple’ mangoes. Future studies should investigate whether excluding surface moisture by bagging or strengthening the fruit skin by the application of gibberellins are effective in controlling russeting in ‘Apple’ mango.

## Supporting information

S1 File This is the excel file containing the data presented in Figs 1–10 and Table 1. Where regression lines were fitted, parameter estimates of regression equations and coefficients of determination are provided.
(XLSX)
